# Parental Alienation Syndrome as a Consequence of Paranoid Contagion or Shared Psychosis

**DOI:** 10.1192/j.eurpsy.2022.1823

**Published:** 2022-09-01

**Authors:** A. Ruiz-Tristan, J. Urricelqui, V. Gomez-De-Las-Heras, V. Crossley

**Affiliations:** 1 Hospital 12 de Octubre, Psychiatry, Madrid, Spain; 2 Hospital Infanta Leonor, Psychiatry, Madrid, Spain

**Keywords:** paranoid, Psychosis, alienation

## Abstract

**Introduction:**

Parental Alienation Syndrome (PAS) was proposed by Richard A. Gardner in 1985. It is assumed to occur in some distressing marriage break-ups, when a parent “brainwashes” his children so they reject the other parent in an unjustified way. But, is it the result of a conscious act as Gardner suggests? Or could it also appear as part of a shared psychosis?

**Objectives:**

To assess the possibility of the appearance of PAS as a consequence of paranoid contagion or shared psychosis.

**Methods:**

We present the case of a 45-year-old patient and her 9-year-old daughter, who is allegedly assaulted by her father during visits, according to both. Mother and daughter continually request attention in the emergency department for this reason, with no obvious injuries. A bibliographic review is carried out on the PAS and shared psychosis. We compare the existing data with our case.

**Results:**

A paranoid cognitive style is observed in the 45-year-old patient, and it is observed that her daughter stops rejecting the father when she spends time separated from her. The contagion of delirium is the nuclear mechanism of shared psychosis. It is known that children with PAS may have distorted memories and incorporate beliefs of others through suggestion. There is also an inverse relationship between the number of visits by the alienated parent and the undervaluation of the child. We have not found any studies linking shared psychosis with PAS.

**Conclusions:**

The existing bibliography on PAS is scarce. The possibility of an existing paranoid contagion mechanism has not been addressed yet.

**Disclosure:**

No significant relationships.

